# 

**Published:** 1822-11

**Authors:** 


### MONTHLY CATALOGUE OF MEDICAL BOOKS.

A Treatise on Dislocations, and on Fractures of the Joints. By Sir Astley Cooper, F.R.S. 1 vol. 4to. with thirty Engravings. 1l. 11s. 6d. boards.

Nichol on Cerebral Structures occurring in Infants. 12mo. 4s.

Nichol's Elements of Pathology. 8vo. 9s. boards.

Bacot's Observations on the Use and Abuse of Friction. 8vo. 2s.

Researches respecting the Medical Power of Chlorine, particularly in Diseases of the Liver; with an Account of a new Mode of applying this Agent. By W. Wallace, M.R.I.A. 8vo. 6s. boards.

Analytic Physiology. By Samuel Hood, M.D. A.B. 8vo. 10s. 6d. boards.

Remarks and Practical Results of Observations on Scirrhus and Cancer; by Antonio Scarpa. Translated from the Italian, with an Appendix, containing an Account of the Sanguineous Tumor of the Breast, with some Observations on the Text, by James Briggs. 8vo. 3s.

Anatomical Plates of the Bones and Muscles, diminished from Albums, for the use of Students in Anatomy and Artists; with explanatory Maps in colours. By Dr. Hooper. 12mo. 7s.

An Address to Parents on the present State of Vaccination in this Country; with an impartial Estimate of the Protection which it is calculated to afford against the Small-pox. By a Candid Observer. 8vo. 3s.

---

### NOTICES TO CORRESPONDENTS.

*Communications have been received from* Mr. Shaw, Dr. Webster, Dr. Ashburner, Mr. Rose, Mr. Benjamin Hutchinson, Dr. Carson, Mr. Cooke, Dr. Baker, Mr. Price, and Mr. Hume.

*We request* Dr. Caron *to accept our thanks for the interesting Cases of Cæsarean Section he has sent us: they shall appear as soon as a coloured Engraving can be executed from the drawing which accompanies the paper.*

Mr. Hutchinson's *polite letter and valuable Communication have been received: we feel much gratified at the prospect of ranking* Mr. H. *among our Correspondents; and he may rest assured that the request he makes shall be faithfully attended to.*

Mr. Bush *has our acknowledgments for his obliging answer to our note.*

*We beg to remind* Mr. Norman *of the hope he has held out of speedily renewing the interesting subject of his paper contained in this Number.*

*The insect described by* Mr. R—, *as vomited by a patient of his, appears to be of the genus* scarabæus. *With regard to the question of their origin, most writers suppose them introduced into the stomach, in the form of ova, in various articles of diet.*

*We request* Professor Naegele, *of Heidelberg, to accept our thanks for his polite letter, and treatise* " De Cephalaematomate."

*We have to acknowledge the receipt of the Books and Pamphlets sent to us by* Dr. Good, Mr. Price, Mr. Swan, Mr. Ward, Dr. Loewe, Mr. Wright, Mr. H. Earle, *and* " A Candid Observer."

